# Longitudinal MRI Evaluation of Intracranial Development and Vascular Characteristics of Breast Cancer Brain Metastases in a Mouse Model

**DOI:** 10.1371/journal.pone.0062238

**Published:** 2013-04-29

**Authors:** Heling Zhou, Min Chen, Dawen Zhao

**Affiliations:** 1 Department of Radiology, University of Texas Southwestern Medical Center, Dallas, Texas, United States of America; 2 Clinical Sciences, University of Texas Southwestern Medical Center, Dallas, Texas, United States of America; City of Hope, United States of America

## Abstract

Longitudinal MRI was applied to monitor intracranial initiation and development of brain metastases and assess tumor vascular volume and permeability in a mouse model of breast cancer brain metastases. Using a 9.4T system, high resolution anatomic MRI and dynamic susceptibility contrast (DSC) perfusion MRI were acquired at different time points after an intracardiac injection of brain-tropic breast cancer MDA-MB231BR-EGFP cells. Three weeks post injection, multifocal brain metastases were first observed with hyperintensity on T_2_-weighted images, but isointensity on T_1_-weighted post contrast images, indicating that blood-tumor-barrier (BTB) at early stage of brain metastases was impermeable. Follow-up MRI revealed intracranial tumor growth and increased number of metastases that distributed throughout the whole brain. At the last scan on week 5, T_1_-weighted post contrast images detected BTB disruption in 160 (34%) of a total of 464 brain metastases. Enhancement in some of the metastases was only seen in partial regions of the tumor, suggesting intratumoral heterogeneity of BTB disruption. DSC MRI measurements of relative cerebral blood volume (rCBV) showed that rCBV of brain metastases was significantly lower (mean  = 0.89±0.03) than that of contralateral normal brain (mean  = 1.00±0.03; p<0.005). Intriguingly, longitudinal measurements revealed that rCBV of individual metastases at early stage was similar to, but became significantly lower than that of contralateral normal brain with tumor growth (p<0.05). The rCBV data were concordant with histological analysis of microvascular density (MVD). Moreover, comprehensive analysis suggested no significant correlation among tumor size, rCBV and BTB permeability. In conclusion, longitudinal MRI provides non-invasive *in vivo* assessments of spatial and temporal development of brain metastases and their vascular volume and permeability. The characteristic rCBV of brain metastases may have a diagnostic value.

## Introduction

Brain metastasis is the most common intracranial malignancy in adults. The prognosis is extremely poor, with a median survival of 4–6 months even with aggressive treatment. Breast cancer is one of the three major primary cancers with a high morbidity of brain metastasis (15–25%) [1]–[3]. Benefited from the routine mammography tests and improved therapeutic options, mortality of breast cancer has been decreasing in the past decade [4]. Advances in chemotherapy and immunotherapy played an important role in treating primary breast cancer as well as its systemic metastasis. However, the incidence of brain metastasis seems to have increased over the past decade, especially within patients undergoing these systematic therapies [5]–[8]. In part, this is due to the fact that most chemotherapeutic agents that show efficacy against systemic disease have poor penetration of blood-brain barrier (BBB). Brain metastases containing an intact BBB are hereby inaccessible to the therapeutics and remain untreated [9]–[11].

Current understandings for vascular development in brain metastases are largely based on invasive histological studies on animal models [12], [13]. Several brain-tropic cancer lines derived from primary melanoma, lung or breast cancer are capable of developing brain metastases upon intracardiac or intracarotid injection [14]–[17]. Using this model, several studies by others have applied molecular tracers to evaluate BTB permeability by measuring their uptake in brain metastases versus normal brain tissues on *ex vivo* brain sections. These studies have shown that BTB is intact at earlier stage of brain metastases, but becomes disruptive while the metastases growing larger [12], [13]. However, histological studies normally require a large number of mice that are killed at different time points after tumor implantation. More importantly, information about temporal development in individual lesions is lacking from histological studies.

*In vivo* imaging promises greater efficiency since each animal serves as its own control and multiple time points can be examined sequentially. Not only intra- and inter-tumoral heterogeneity but also temporal comparison in the same individual lesions can be studied with longitudinal imaging. MRI that has a superb spatial resolution is the most widely used imaging modality for brain tumors of clinical patients. MRI at 1.5 or 3 T has previously been applied by others to study the intracardiac model of brain metastasis of breast cancer MDA-MB231Br (231Br) in mice or rats. Successful detection of multifocal brain metastases and alteration of BTB permeability based on the leakage of MR contrast agents has been reported in their studies [18], [19].

Tumor blood perfusion is another main factor that may affect efficient delivery of chemotherapeutics to brain metastasis. There are several noninvasive imaging modalities available for vascular perfusion assessment. Dynamic susceptibility contrast (DSC) perfusion MRI is one of the most applied MR imaging techniques for brain perfusion measurement [20], [21]. A series of T_2_^*^-weighted images is acquired to capture the rapid passage of gadolinium contrast agent post i.v. injection. The perfusion parameters extracted from decreased signal intensity during the first pass of the contrast are used to calculate cerebral blood volume (rCBV) and flow (rCBF) in a disease site relative to the contralateral healthy brain region. DSC perfusion MRI has been widely used for noninvasive assessment of tumor vascularity in both preclinical and clinical settings [22]–[24]. However, there are currently no studies that have assessed vascular perfusion and their changes with intracranial development of brain metastases. The characteristics of the 231Br model containing multifocal brain lesions that are widespread throughout the whole mouse brain present a technical challenge. To facilitate intertumoral rCBV comparison within and between individual animals, in contrast to the widely-used single normal reference, we have developed a novel approach that utilizes multiple normal references contralateral to the tumor lesions.

In this study, we have applied a high field 9.4 T MRI system to monitor longitudinal development of brain metastases based on T_2_-weighted images after intracardiac inoculation of breast cancer MDA-MB231Br cells. Along with intracranial tumor growth, changes in BTB and tumor vascular volume, rCBV were evaluated by longitudinal T_1_-weighted post contrast images and DSC perfusion-weighted MRI, respectively. Comprehensive analysis was performed to study correlations between tumor volume, BTB disruption and rCBV in individual metastases.

## Materials and Methods

### Cell Preparation

Brain-tropic human breast cancer MDA-MB-231/BR-GFP cell line (231-BR) was previously described [14], [15]. The 231-BR cells (kindly provided by Dr. Steeg, NCI) were incubated in Dulbecco’s modified Eagle’s medium (DMEM) with 10% FBS, 1% L-Glutamine and 1% penicillin-streptomycin at 37°C with 5% CO_2_. Once 80% confluence was reached, the cells were harvested, and suspended in serum-free medium.

### Breast Cancer Brain Metastasis Model

All animal procedures were approved by the Institutional Animal Care and Use Committee of University of Texas Southwestern Medical Center. Female nude mice (n  = 9; BALB/c nu/nu, 6–8 weeks old; NCI, Frederick, MD) were anesthetized with inhalation of 2% isoflurane. 2×10^5^ 231-BR cells (in 100 µl of serum free medium) were injected directly into the left ventricle of a mouse heart under the imaging guidance of a small animal ultrasound (Vevo 770, VisualSonics; Toronto, Canada).

### Longitudinal MRI Studies

#### MRI monitoring of the initiation and development of intracranial tumors

MRI was initiated two weeks after tumor implantation and repeated once a week for up to three weeks. Animals were sedated with 3% isoflurane and maintained under general anesthesia (1.5% isoflurane). Animal body temperature and respiration were monitored and maintained constant throughout the experiment. MR measurements were performed using a 9.4 T horizontal bore magnet with a Varian INOVA Unity system (Palo Alto, CA). A tail vein of mouse was catheterized using a 27 G butterfly for Gd-DTPA (Magnevist®; Bayer HealthCare, Wayne, NJ) contrast agent administration. High resolution multi-slice (14 slices with 1 mm-thick, no gap) T_1_- and T_2_-weighted coronal images, covering from the frontal lobe to the posterior fossa, were acquired with the following parameters: T_1_-weighted images: spin echo multiple slice (SEMS), TR/TE  = 400 ms/20 ms, matrix: 256×256, FOV 20×20 mm, resolution: 78×78 µm^2^ in plane. T_2_-weighted images: fast spin echo multiple slice (FSEMS) sequences, TR/TE  = 2500 ms/48 ms, 8 echo trains, matrix: 256×256, FOV 20×20 mm, resolution: 78×78 µm^2^ in plane. We determined tumor size on T_2_-weighted images by manually outlining the enhancing portion of the mass on each image by using MatLab (Mathworks, Natick, MA) programs written by us. With high spatial resolution, hyperintense lesions can be identified as small as 310 µm in diameter. Since the metastases in this study were very small and development of necrosis and edema was minimal, the hyperintense lesion on T_2_-weighted images truly represented the tumor mass. Given most of the tumor diameters were smaller than the slice thickness (1 mm), the tumor size was presented as in plane area rather than the volume.

#### Dynamic Susceptibility Contrast (DSC) perfusion MRI

**Figure 1:**
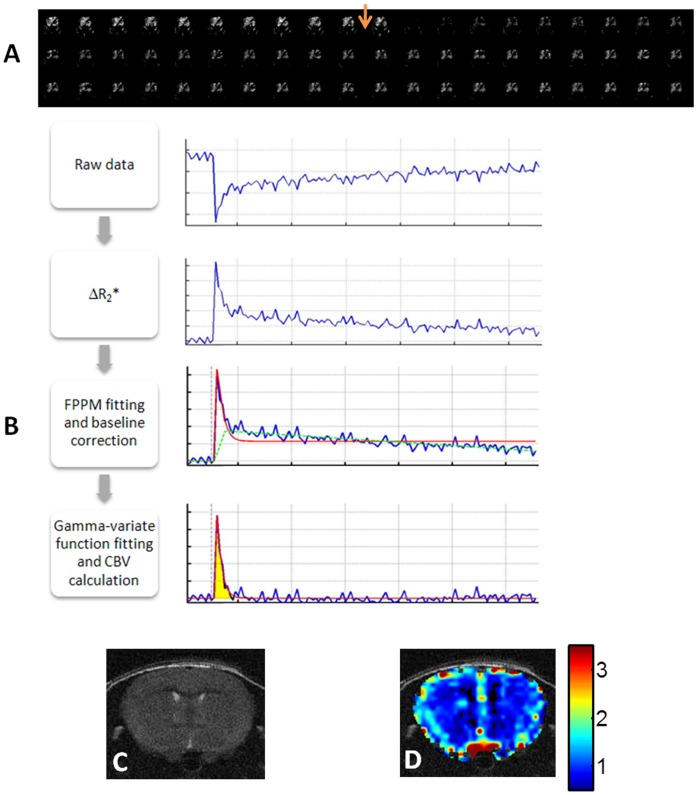
Dynamic susceptibility contrast (DSC) MRI of rCBV. **A.** A series of T_2_^*^-weighted images of a mouse brain was acquired before and after a bolus injection of the contrast agent, Gd-DTPA via a tail vein (arrow). Immediately after the injection, significant loss in signal intensity was observed, which gradually recovered to the baseline level 5 mins later. **B**. The flowchart illustrates the data process of DSC MRI to generate rCBV map. Raw data of DSC MRI signal versus time curve was first plotted, depicting the first pass of the contrast agent via the brain as the dip on the curve. The ΔR_2_^*^ was then calculated from the signal time course. FPPM was applied to determine the general trend of ΔR_2_^*^ and a three-segment baseline was generated. Finally, Gamma-variate fitting was used to correct ΔR_2_^*^, and the area under the bolus was calculated, which is proportional to CBV. **C**. An anatomic T_2_-weighted image was obtained from a normal mouse brain. A color-coded rCBV map generated from DSC MRI was overlaid on the T_2_-weighted image showing symmetric distribution of rCBV between the two hemispheres (**D**).



Once brain lesions were identified on T_2_-weighted images, DSC MRI was performed on 4 of the slices containing most of the metastases. A series of gradient echo multiple slice (GEMS) T_2_^*^-weighted images over 5 mins was acquired before and after a bolus injection of Gd-DTPA contrast (0.1 mmol/kg body weight) via a tail vein. DSC MRI parameters: TR/TE  = 27 ms/4 ms, FA  = 20°, matrix: 64×64, FOV 20×20 mm, number of slices  = 4 with 1 mm thick. As illustrated in Fig. 1, raw data of signal intensity were extracted from the image series on a voxel-by-voxel basis and plotted into a time course curve. ΔR_2_^*^ was calculated using equation:
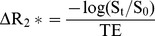
(1)where St is the signal intensity (SI) of each time point, S_0_ is the mean SI of baseline, and TE is the echo time. First-pass pharmacokinetic modeling (FPPM) fitting [25] was applied to ΔR_2_^*^ curve to detect the starting and the ending points of the bolus. The equation used for this step is as follows:
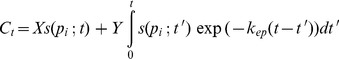
(2)Where

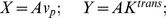



And the bolus shape function is defined as:
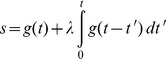
Where



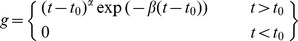



Baseline correction was separated into three parts. Pre-bolus baseline was set to zero. Then, a linear fit was applied from this point to the end of the data to obtain post bolus baseline, and connection of these two segments served as the under-bolus baseline. Subtraction of ΔR_2_^*^ with baseline obtained in previous step provided a corrected ΔR_2_^*^. CBV was calculated from the gamma-variate fitting applied to the corrected curve and the area under the fitted curve was proportional to CBV [23]. Gamma fitting was obtained using the following equation:
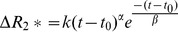
(3)


ROIs of brain metastases were drawn based on the high resolution T_2_-weighted images. The corresponding contralateral normal ROIs were selected symmetrically to the tumor ROIs according to the distinct landmarks in brain, such as ventricles, hippocampus and the interfaces of different anatomical structures. In cases where the contralateral sites appeared abnormal, neighboring regions in the same anatomical structure were selected instead. The mean CBV value of all the normal ROIs in each animal served as the reference. rCBV for both individual metastases and the normal brain was determined by normalizing the CBV to the reference CBV. Analysis was performed on a home written MATLAB program. A color-coded rCBV map was obtained and overlaid on the corresponding T_2_-weighted image.

#### Brain Tumor Barrier (BTB) permeability

T_1_-weighted spin echo multislice images were immediately acquired after DSC MRI, about 5 mins post contrast injection to evaluate BTB permeability. A non-permeable metastasis is isointense, while a permeable metastasis appears hyperintense to surrounding brain tissue on T_1_-weighted post contrast image.

### Histological and Immunohistochemical Studies

Animals were sacrificed immediately after the last MR follow-up and the brain was dissected and frozen for preparation of cryosections. H&E staining was performed on coronal brain sections (10 µm), while their adjacent sections were used for immunohistological staining. Vascular endothelium was stained using a rat anti-mouse CD31 antibody (Serotec Inc., Raleigh, NC) followed by Cy3-labeled goat anti-rat IgG (Jackson Immunoresearch Laboratory, West Grove, PA). Fluorescence images of tumor cells (green, GFP) and vasculature (red) were captured on the same microscopic field using a Coolsnap digital camera mounted on a Nikon microscope and analyzed with MetaVue software (Universal Imaging Corporation). Microvascular density (MVD) was calculated as follows: mean number of red vessels/mean area of green tumors. A total of twelve metastases and their contralateral normal brain tissues in 3 tumor-bearing mouse brains were evaluated.

### Statistical Analysis

Statistical analysis was conducted using R (R Development Core Team, 2012), a language and environment for statistical computing. A natural log transformation was applied to all the rCBV values to achieve normal distribution before Student’s t-test was performed for significance analysis. The statistical analysis of correlation was based on Regression and Bivariate plots (SAS Inst. Inc., Cary, NC). Two sample paired Student’s t-test was performed to evaluate the significant difference of rCBV or MVD of tumor and its contralateral reference tissue. After dividing tumors into two groups based on the permeability status, comparison of rCBV or tumor size between these two groups was evaluated using unpaired t-test. Paired t-test was performed for longtitudinal studies to assess the changes of individual lesions over time.

## Results

**Figure 2:**
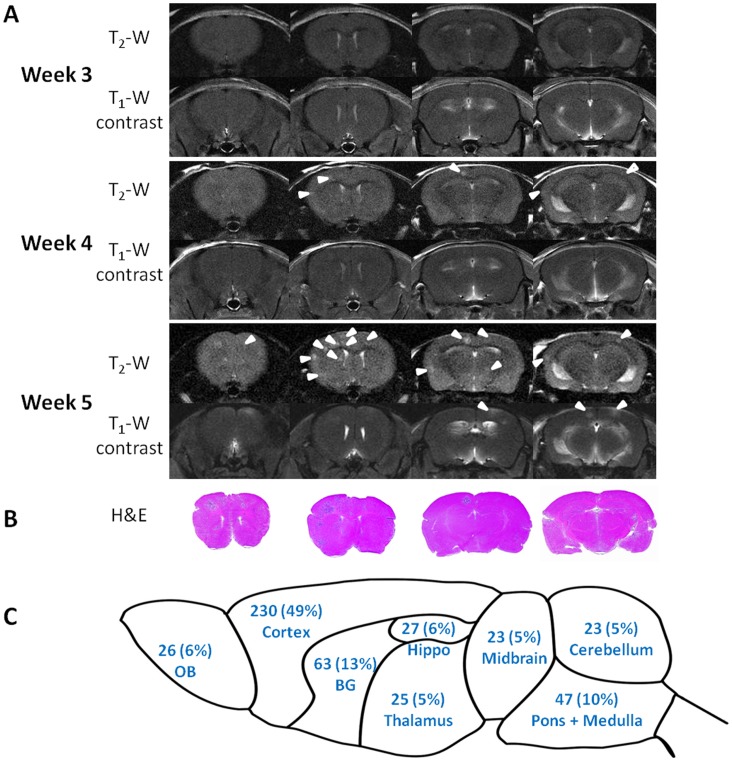
Longitudinal MRI monitoring of the initiation and development of intracranial brain metastases. **A.** MRI scans of the whole mouse brain were initiated 3 weeks after intracardiac injection of MDA-MB231Br cells and repeated once a week for 2 weeks. Four consecutive coronal MRI sections of a representative mouse brain showed no apparent intracranial lesions on T_2_-weighted images. However, follow-up images at week 4 identified multiple lesions with hyper-intensity on T_2_-weighted images (arrowhead), but none of them was enhanced on T_1_-weighted post contrast images. An increased number of lesions (arrowheads) appeared on the images at week 5, only a few of which (arrowheads) were enhanced post Gd-DTPA. **B.** Corresponding histological sections of H&E staining showed a good correlation with MRI. **C.** MRI evaluation of a total of 464 metastases in 9 mice brains indicated that these metastases distributed through the whole mouse brain with a higher incidence in the brain cortex (49%). Note: OB: Olfactory bulb; BG: Basal ganglia; Hippo: Hippocampus.

**Figure 3:**
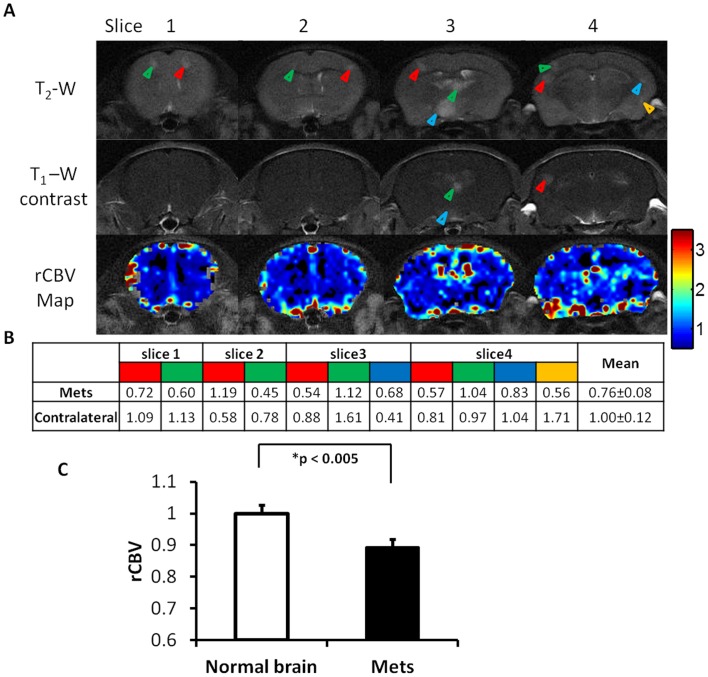
Significantly lower rCBV in brain metastases than contralateral normal brain. **A.** Four weeks after intracardiac injection of 231Br cells, T_2_-weighted MRI revealed multiple high signal intensity lesions (arrowheads) on four consecutive coronal sections of a representative mouse brain. Only a few of the lesions (arrowheads) were enhanced on T_1_-weighted post contrast images, one (blue arrowhead in the MRI section 3) of which showed partial enhancement, indicating intratumoral heterogeneity of BTB disruption. rCBV maps of the four sections were generated and overlaid on the T_2_-weighted images. **B.** The rCBV values of the metastatic lesions and their contralateral normal brain were obtained and summarized in the table. Note the color presented in the table coincides with the color of arrowhead on each of the MR images. Most of metastatic lesions had lower rCBV values than their contralateral counterparts of normal brain. **C.** Statistical analysis of rCBV in a total of 212 lesions of 9 animals obtained from the last follow-up MRI showed significantly lower rCBV of the metastatic tumors with a mean value of 0.89±0.03 (s.e.), compared to the contralateral normal brain (mean  = 1.00±0.03; p<0.005).

**Figure 4:**
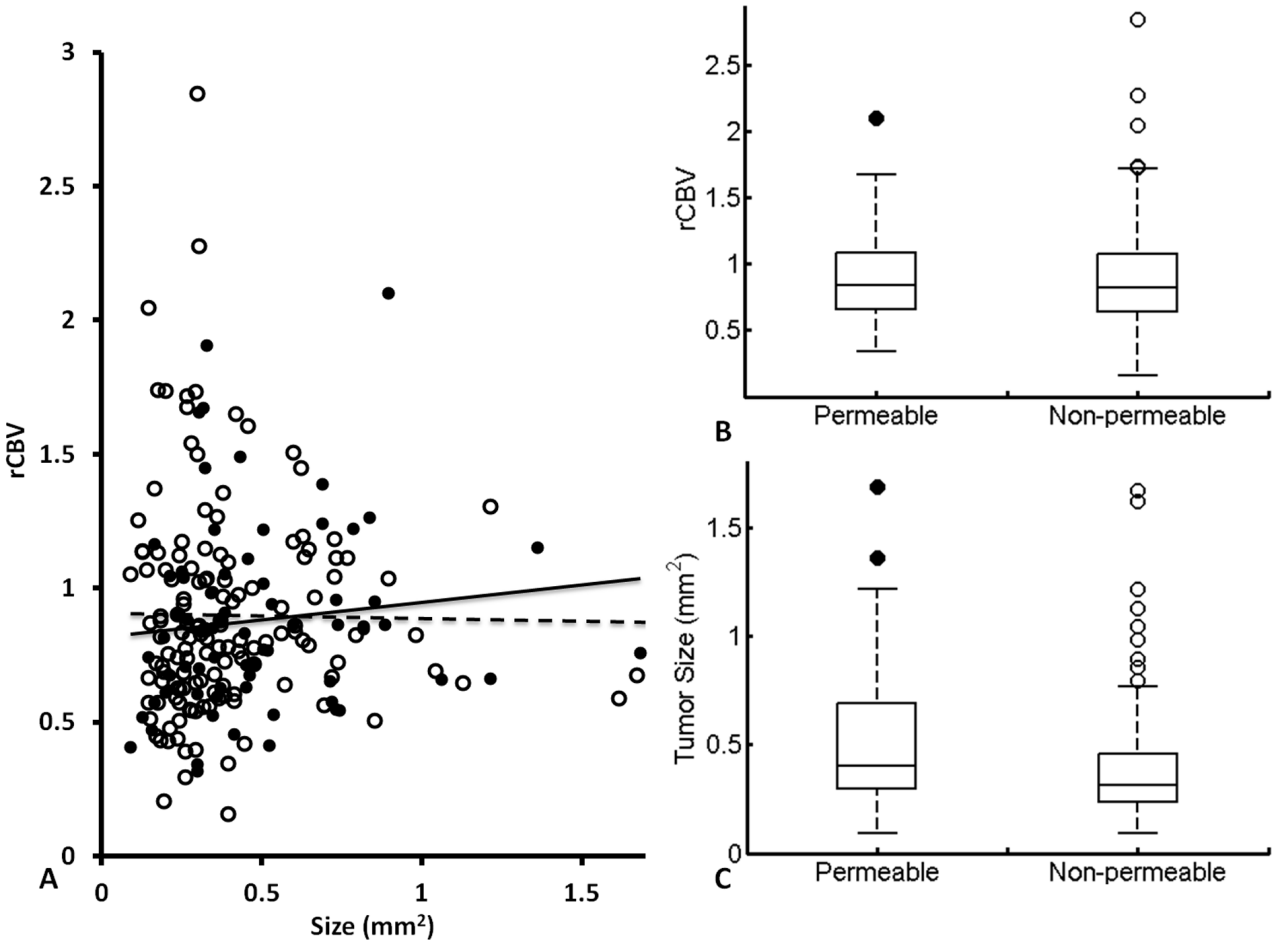
Lack of correlation between rCBV, tumor size and permeability of brain metastases. Based on T_1_-weighted contrast enhanced MRI, the 212 metastases studied by DSC MRI were separated into the permeable (enhanced, n  = 70) and non-permeable (not enhanced, n  = 142) group. **A.** A plot of rCBV versus individual tumor size showed no correlation in either the permeable (filled; R^2^ = 0.01) or non-permeable group (empty; R^2^<0.02). **B.** The rCBV values of the permeable lesions (median  = 0.84, ranging from 0.34 to 2.10) were not significantly different from those of the non-permeable ones (median  = 0.82, ranging from 0.16 to 2.84; p>0.1). **C.** Further comparison found no significant difference in tumor size between the permeable (mean  = 0.49±0.11 mm^2^) and the non-permeable (mean  = 0.47±0.14 mm^2^; p  = 0.1) metastases.

**Figure 5:**
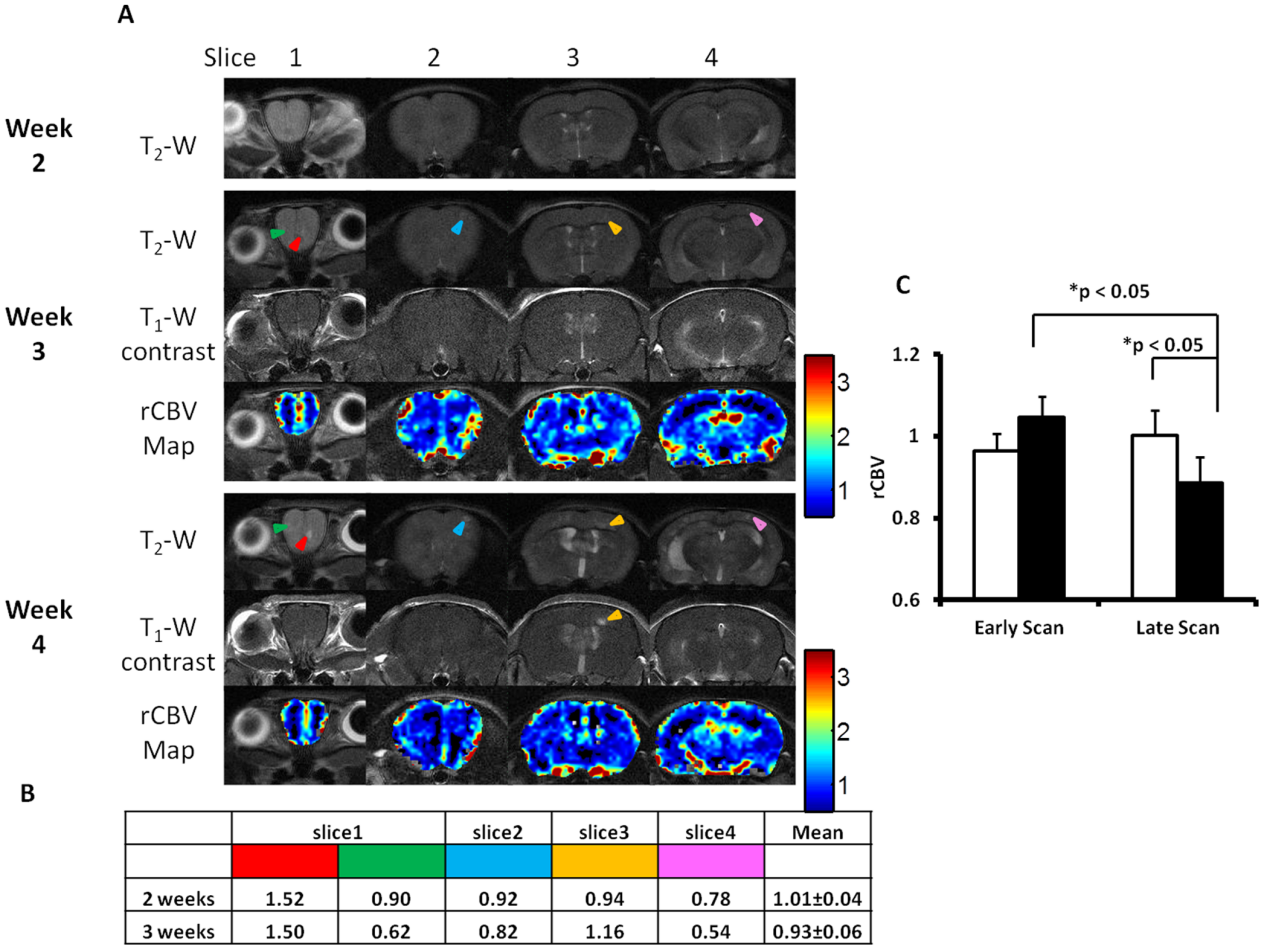
Longitudinal MRI study of changes in BTB permeability and rCBV of brain metastases. **A.** Longitudinal MRI of a representative mouse brain was initiated 2 weeks after intracardiac injection of 231Br cells. At week 3, five small metastases (arrowhead) were identified on four consecutive T_2_-weighted coronal images. At week 4, many more lesions appeared on T_2_-weighted coronal images, while all the 5 lesions seen on week 3 were found to increase in size (arrowhead). Changes in BTB permeability and rCBV were then evaluated for these five lesions. There was initially no contrast enhancement seen in the five tumors at week 3, indicating an intact BTB. All the tumors except one (yellow arrowhead) still kept BTB intact at week 4. rCBV maps were created and rCBV values of the tumors were presented in the table (**B**). **C.** A total of 32 lesions in 5 animals were seen on both scans of weeks 3 and 4. rCBV of brain metastases (solid) was initially similar to that of contralateral normal brain (open; mean  = 1.05±0.05 (se) vs. 0.96±0.04), but decreased significantly (p<0.05) and became significantly lower as compared to their contralateral normal brain in the late scan (mean  = 0.88±0.06 vs. 1.00±0.06; p<0.05).

Ultrasound imaging-guided left ventricular injection of brain-tropic breast cancer 231BR cells ensured the accuracy so that every animal in this study developed brain metastases, as compared to a successful rate of 50% with the manual injection used in our previous study. Longitudinal MRI monitoring was initiated 2 weeks post injection and repeated once a week for up to 3 more weeks (Fig. 2). With the high spatial resolution of T_2_-weighted images, metastatic lesions, appearing as hyperintense, became visible in mouse brain 3 or 4 weeks post injection; the minimum detectable tumor was ∼310 µm in diameter, whereas all of these early stage metastases were isointense on T_1_-weighted post contrast images (Figs. 2, 3, 4, 5). Follow-up scans revealed increased tumor size and appearance of new lesions (Figs. 2A and 5A), which correlated well with H&E staining (Fig. 2B). A total of 464 metastases, with a size ranging from 0.09 mm^2^ to 1.7 mm^2^, in 9 mice were depicted on T_2_-weighted images of the last MR follow-up at week 5. These metastases distribute throughout the mouse brain with a higher incidence in the cerebral cortex (49%), whereas the least number in the thalamus, midbrain or cerebellum (5%; Fig. 2C). Of these metastases, 160 (34%) lesions were enhanced on T_1_-weighted post contrast images, indicating a locally disrupted BTB (Figs. 2A, 3A, 4 and 5A). However, enhancement in some of the metastases was only seen in partial regions of the tumor (Fig. 3A), suggesting intratumoral heterogeneity of BTB disruption.

Dynamic susceptibility contrast (DSC) MRI, based on a bolus injection of contrast agent Gd-DTPA into a tail vein, was applied for rCBV measurement (Fig. 1A). The first pass of the contrast via brain vasculature was clearly depicted as the dip region in the signal intensity time cure (Fig. 1B). A color-coded rCBV map of a normal mouse brain, projected on the T_2_-weighted image, showed the high level of symmetry between hemispheres and significant higher rCBV in the cortical regions (Fig. 1C and D). For brain metastases-bearing mice (n  = 9), 212 of the 464 metastases, identified at the last scan, were subject to rCBV measurements and compared with their contralateral normal brain. As shown in Fig. 3, the metastatic lesions were found to have significantly lower rCBV, compared to their contralateral normal brain (mean  = 0.89±0.03 (s.e.) vs. 1.00±0.03 (s.e.), p<0.005; Fig. 3C). It is important to notice marked heterogeneity in rCBV between individual metastases, ranging from 0.16 to 2.84 (Figs. 3, 4, 5). This is also true for rCBV in normal brain, as demonstrated in Fig. 1. Thus, it is necessary to compare rCBV between a pair of a specific metastases and its contralateral normal brain. Further analysis showed no significant correlation between rCBV and tumor size (R^2^<0.02; Fig. 4A). When these metastases were separated into the permeable (n  = 70) and non-permeable (n  = 142) group, determined upon whether it was enhanced by T_1_ contrast agent, the data showed that neither rCBV (mean  = 0.88±0.04 vs. 0.90±0.03; p>0.1; Fig. 4B) nor tumor size (mean  = 0.49±0.11 mm^2^ vs. 0.47±0.14 mm^2^; p>0.1; Fig. 4C) significantly differed between the two groups.

Longitudinal MRI studies allowed *in vivo* non-invasive evaluation of tumor growth and changes in BTB permeability and rCBV of individual brain metastases. Thirty two metastases that were identified from 5 of the 9 mouse brains in the scans of week 3 were followed a week later. As shown in a representative mouse brain in Fig. 5, five small hyperintense metastases first appeared on T_2_-weighted images at week 3. All the 5 lesions grew larger, along with many other new lesions becoming visible in the following week’s scan. All the 5 lesions showed no enhancement on T_1_-weighted contrast images at week 3, only one of them became enhanced at week 4. DSC MRI found that rCBV values decreased in 4 of the 5 metastases (Fig. 5B). For the total of 32 metastases, rCBV values were initially similar to those of their contralateral normal brain (mean  = 1.05±0.05 vs. 0.96±0.04), but decreased significantly and became significantly lower than those of their contralateral normal brain in the late scan (mean  = 0.88±0.06 vs. 1.00±0.06, p<0.05; Fig. 5C).

**Figure 6:**
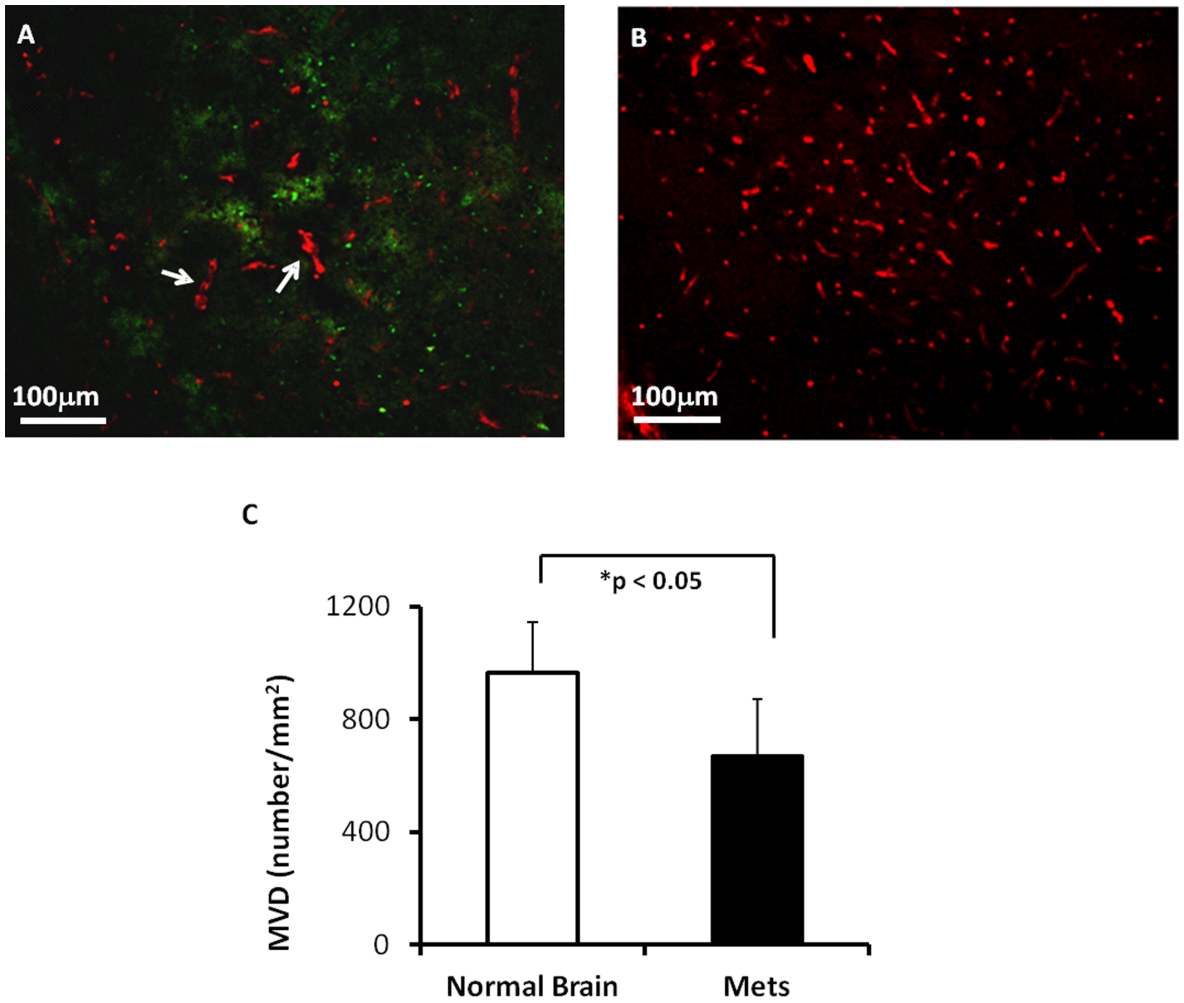
Immunohistochemical study of microvascular density (MVD) in brain metastases. **A.** Anti-CD31 staining was performed on a brain section bearing metastases. A cortical lesion (∼ 600 µm in diameter) was depicted with green fluorescence (GFP). Microvessels (red) within the lesion appeared less dense, as compared to abundant fine vessels in the contralateral normal brain tissues (**B**). Some of the tumor vessels were irregular in shape and larger in diameter (arrow). **C.** Quantitative data of MVD showed a significantly lower MVD in brain metastases versus contralateral normal brain (mean  = 669±201/mm^2^ vs. 965±177/mm^2^; p<0.05).

Immunohistochmical staining of vascular endothelium (CD31) showed that MVD was 669±201/mm^2^ within the metastatic lesions (n  = 12), which was significantly lower than that of the contralateral normal brain (965±177/mm^2^; p<0.05; Fig. 6). Moreover, in contrast to the normal brain comprising of regularly-shaped micro-vessels, the irregular and dilated vessels were often seen for tumor vessels (Fig. 6).

## Discussion

In the present study, we have demonstrated the utility of longitudinal MRI to evaluate intracranial growth and vascularity of breast cancer brain metastases in a mouse model. Using the 9.4 T MRI, high resolution T_2_-weighted images enabled the detection of multifocal tumor initiation at a diameter of as small as 310 µm. Longitudinal monitoring of BTB permeability based on T_1_-weighted contrast enhanced images revealed that BTB in early-stage (week 2 or 3) brain metastases were exclusively impermeable; even at the late stage (week 4 or 5), T_1_ contrast enhancement was only found in a small proportion (34%) of brain metastases, indicating that the BTB is still intact in the majority of the metastases (Figs. 2, 3 and 5). This observation is in good agreement with a recent MRI study of the 231BR brain metastases mouse model. In that study, Percy et al observed no contrast enhancement for brain metastases by day 20, while 28% of the metastases by day 30 appeared hyperintense on T_1_-weighted post contrast images [18]. The MRI data are consistent with histological studies conducted previously by others. Zhang and colleagues administered fluorescent dye, sodium fluorescein systemically into the mice bearing brain metastases to study BBB permeability. The microscopic observations on brain sections showed differential permeability of the dye among the metastases, of which lesions smaller than 0.2 mm^2^ had intact BBB, while larger metastases were leaky because of tumor angiogenesis and/or central necrosis [12]. Although our data of the current study indicated that the mean tumor size of permeable metastases was larger than that of the non-permeable ones, there was no significant difference in tumor size between the two groups (Fig. 4C). Similarly, lack of correlation between tumor size and BBB disruption was reported in a recent study by Lockman et al., suggesting other factors may also be involved in this dynamic process [13].

On many occasions, enhancement on T_1_-contrast images was only seen in partial regions of the tumor (Fig. 3C), implicating inhomogeneous disruption of BTB. This finding concurs with a recent study of biodistribution of anti-cancer drugs in brain metastases [13]. Lockman and colleagues assessed the uptake of radio-labeled paclitaxel or doxorubicin in brain metastases of MDA-MB231Br-Her2 mouse model. Heterogeneous intratumoral distribution of the chemotherapeutics was clearly visualized on *ex vivo* brain sections by phosphorescence. Even in the most permeable metastases, the drug concentrations were far below that in visceral metastases [13]. Taken together, all these data support the notion that systemic anti-cancer therapeutics has limited utility in treating brain metastases.

Despite its small molecular weight (MW ∼ 500), the hydrophilic MRI contrast, Gd-DTPA is found not to penetrate across the intact BBB and is thus suitable for BBB permeability study. Many strategies to overcome this barrier have been exploited to facilitate the delivery of effective anti-cancer therapeutics into brain tumors, i.e, hemispherical opening of the BBB using high-concentration intra-carotid injections of mannitol or temporary disruption of localized BBB by high-intensity focused ultrasound [26], [27]. Thus, it is critical to develop a means to enable non-invasive evaluation of BBB damage after exposure to intervention in order to predict drug delivery. Indeed, Treat et al recently reported a linear correlation between T_1_ signal intensity post Gd-DTPA and doxorubicin concentrations in brain regions after BBB damage induced by sonication [28].

Perfusion MRI has been widely used to provide important diagnostic and prognostic information on pathological conditions. Arterial spin labeling (ASL) MRI, utilizing magnetically tagged arterial blood as endogenous contrast has proven feasible in quantitative measurements of rCBF and rCBV in clinical studies [29], [30]. However, the low sensitivity and poor signal to noise ratio (SNR) of ASL perfusion MRI limits its application to mouse brain. Dynamic contrast susceptibility (DSC) MRI acquired after infusion of MRI contrast agents is another technique to measure rCBV. DSC MRI has been widely applied to study microvasculature and hemodynamics in brain tumors. Recent studies have correlated DSC MRI of rCBV with histological grade and degree of neovascularization in human glioma [23], [31], [32]. In a preclinical mouse glioma study, a positive correlation between rCBV and tumor microvascular density (MVD) was found [24]. However, little is known about rCBV abnormality of brain metastases, in particular, its variation among individual brain metastases as well as its alteration with tumor development.

Our data showed marked heterogeneity of rCBV for both normal brain and metastatic lesions (Figs. 1 and 4). To acquire an rCBV value of a lesion, a region of normal brain needs to be chosen to serve as a reference. Previously published clinical or preclinical works on vascular perfusion of a solitary brain tumor have utilized either a region of white matters or the normal brain contralateral to the tumor as a single reference [23]. Tthe intracardiac brain metastasis model used in this study developed multiple intracranial lesions. As shown in Fig. 2, these multifocal metastases distributed well throughout the whole mouse brain. It is difficult to define a specific region of normal brain that can be used as a common reference for each individual case. Moreover, given the marked heterogeneity in vascular perfusion of normal brain, we applied multiple normal regions that locate contralaterally to the individual metastases as the references. The mean CBV value of all the normal ROIs in each animal was used to serve as the reference for both metastatic lesions and normal brain regions. In cases where the contralateral sites appeared abnormal, neighboring regions in the same anatomical structure were selected instead. As shown in Fig. 1D, using this approach, the reliable rCBV map was generated in a normal mouse brain. We believe that this approach can significantly minimize the individual variation and facilitate a comparison of rCBV between individual animals.

DSC MRI measurement of rCBV revealed significantly lower rCBV of brain metastases than that of contralateral normal brain (p<0.005). The rCBV data were in good agreement with histological findings that MVD within the metastases was significantly lower than that of contralateral normal brain. Fidler’s group has documented in their published studies that the MVD within the metastases was 20 times lower that in the surrounding normal brain [12]. Unlike the robust angiogenesis observed in primary high grade glioma, vessel sprouting, the characteristic of angiogenesis, is rarely seen in brain metastases. Instead, dilation of blood vessel lumen, as shown in fig. 6, and reported elsewhere in both the experimental brain metastases and surgical specimens of human lung cancer brain metastases, is considered as a result of the division of endothelial cells [12], [33]. The characteristic rCBV may have a diagnostic value for brain metastases to differentiate them from those highly vascularized and perfused malignant gliomas.

Longitudinal MRI allows rCBV of individual brain metastases to be examined over time. Intriguingly, our data showed that rCBV of individual metastases at early stage was similar to, but became significantly lower than that of their healthy counterparts at the late stage of tumor development (Fig. 5C; p<0.05). By using multiphoton laser scanning microscopy and a mouse cranial window model, Kienast et al. followed in real time brain metastases formation from lung cancer and melanoma in mouse brain. After extravasation, the metastatic cells grow along the preexisting normal brain vessels [34]. In another study, Kusters et al. showed that a melanoma brain metastasis could grow up to 3 mm through co-opting preexisting blood vessels without induction of an angiogenic switch [35]. All these data indicate that there is no angiogenic compensation for the tissue volume increase at the lesion site, resulting in a lower MVD and rCBV, as compared to normal brain. For the pooled data, however, we found that rCBV was not correlated with either the size or permeability of metastasis (Fig. 4A and B), implicating that the intertumoral heterogeneity of rCBV may result from regional variation in vascularity in normal brain where metastases locate.

In summary, we have applied 9.4 T MRI to study brain metastases formation after intracardiac injection of breast cancer MDA-MB231Br-GFP cells into mice. High resolution T_2_-weighted MRI enables the detection of multifocal metastases at early stage. MRI contrast, Gd-DTPA based T_1_-weighted contrast enhanced MRI and T_2_^*^-weighted DSC MRI allow non-invasive characterization of vascular permeability and blood volume during intracranial development of brain metastases. Significant lower BTB permeability and vascular volume in brain metastases than normal brain underscore the urgent need to develop brain permeable drugs or a means to alter the BTB permeability in order to achieve therapeutic concentrations of anti-cancer therapeutics.
